# Drawing the Line

**Published:** 1892-07

**Authors:** Hayes C. French

**Affiliations:** 114 Geary St., San Francisco


### DRAWING THE LINE.

114 GEARY ST., SAN FRANCISCO, April 25th, 1892.

EDITOR OF THE HOMŒOPATHIC PHYSICIAN:

I could not keep house without your Journal. I do not like the Phariseeism of some of your leaders when I see so many of their blunders. But when a *nominal* homœopath comes at me with some new allopathic treatment for headache I draw the line there, and say let us have one pure advocate of pure Homœopathy.

I have the January number; please send me the balance for this year.

Yours,

HAYES C. FRENCH.

